# Postprandial Glycemic Responses to Rice Porridge Versus Cooked Rice in Healthy Young Adults: A Randomized Controlled Crossover Study

**DOI:** 10.7759/cureus.112070

**Published:** 2026-07-04

**Authors:** Kosuke Kodama, Atsuko Kayashita, Niina Tsuji, Haruko Kakehashi, Miki Tamura, Jun Kayashita

**Affiliations:** 1 Nutrition, Graduate School of Comprehensive Scientific Research, Prefectural University of Hiroshima, Hiroshima, JPN; 2 Sports and Health, Meio University, Nago, JPN; 3 Health Sciences Studies, Prefectural University of Hiroshima, Hiroshima, JPN; 4 Nutrition, Kobe Gakuin University, Kobe, JPN

**Keywords:** cooked rice, food form, gastric emptying, interstitial fluid glucose, rice porridge

## Abstract

Background: For individuals with dysphagia, rice porridge (RP) is a standard staple. However, its effect on postprandial glycemia compared to cooked rice (CR) - even with matched carbohydrate content - remains unclear. This study aimed to evaluate the impact of food form (CR vs. RP) on interstitial fluid glucose (IFG) levels using flash glucose monitoring (FGM).

Methods: A randomized crossover study was conducted with 31 healthy university students (mean age 20.3 years). Subjects consumed either CR or RP, both standardized to 40.7 g of carbohydrates. IFG was measured using the FreeStyle Libre flash glucose monitoring system (Abbott Diabetes Care, Alameda, CA, USA) every 15 minutes for 120 minutes post-ingestion. Mastication was limited to 10 strokes per mouthful to simulate impaired oral processing. Primary outcomes were the incremental area under the curve (iAUC0-120) and time-specific IFG levels.

Results: Compared to CR, RP showed significantly higher IFG levels at 30, 45, and 60 minutes post-ingestion (p < 0.01). The iAUC was significantly greater for RP across all time intervals (0-30, 0-60, 0-90, and 0-120 min) (p < 0.05). No significant difference was observed in subjective fullness (Visual Analogue Scale (VAS)) at 120 minutes (p = 0.1).

Conclusions: In healthy young adults, RP induces a more rapid and higher postprandial glucose response than CR despite identical carbohydrate content. This suggests that the increased surface area and gelatinization in porridge facilitate starch hydrolysis. Clinical nutritional management for patients requiring texture-modified diets should account for these glycemic differences.

## Introduction

The prevalence of diabetes mellitus continues to rise globally. In Japan, the 2019 National Health and Nutrition Survey reported that approximately 19.7% of men and 10.8% of women are suspected of having diabetes mellitus [1]. This prevalence increases significantly with age. Concurrently, aging is associated with a decline in oral functions; approximately 36.6% of individuals aged 70 and older report difficulty chewing firm foods, and 27.2% experience choking during fluid intake [1]. Consequently, a substantial number of elderly individuals face the dual challenge of impaired masticatory/swallowing functions and impaired glucose tolerance.

For individuals with dysphagia, modifying food texture is essential for safe swallowing. While cooked rice (CR) is the staple of the Japanese diet, those with impaired oral processing often consume rice porridge (RP) (soft-CR with higher water content). Although the glycemic index (GI) of rice is a well-known indicator of postprandial blood glucose response, it is reported to vary significantly depending on cooking methods and physical form, even when using the same ingredients [2-4]. Rice is no exception; previous studies have shown that differences in heating time and water-to-rice ratios result in different GI values [5]. However, research directly comparing standard CR and RP - standardized for carbohydrate content - is scarce. Therefore, the extent to which these structural differences influence postprandial glycemic responses remains poorly defined.

Furthermore, the timing of meals, particularly the presence or absence of breakfast, is known to significantly affect daily glucose metabolism and clock gene expression [6,7]. To account for these physiological variations and the "second-meal effect," a controlled experimental setting is required to isolate the impact of food form itself.

This study aimed to evaluate the differences in postprandial interstitial fluid glucose (IFG) responses between CR and RP using a flash glucose monitoring (FGM) system (FreeStyle Libre; Abbott Diabetes Care, Alameda, CA, USA). While the ultimate target of this research is the elderly with impaired oral functions, conducting such tests in this population poses risks such as aspiration. Therefore, we recruited healthy young adults and strictly limited their mastication to simulate the impaired oral processing of the elderly while ensuring participant safety. We hypothesized that even with identical carbohydrate loads, the higher water content and advanced starch gelatinization in RP would lead to a more rapid and higher glycemic response compared to CR.

## Materials and methods

Subjects

The study initially recruited 38 healthy university students (aged 18-25 years). The study was conducted during a two‑week period between October and November 2020. Exclusion criteria included: (1) BMI >30 kg/m^2^, (2) current insulin therapy, (3) history of gastrointestinal surgery, (4) pregnancy or lactation, (5) cognitive impairment, (6) allergy to test meal ingredients, (7) age < 18 years, and (8) diagnosis of glucose intolerance within the past year. Based on data from similar previous studies, the required sample sizes calculated using G*Power (Heinrich-Heine-Universität Düsseldorf, Düsseldorf, Germany) were 15 and 18, indicating that the sample size of the present study satisfies the sufficient conditions [8,9]. In the present study, 31 participants were included, which is relatively larger than those in prior studies. Therefore, the sample size of this study is considered appropriate in comparison with the existing literature and adequate from the perspective of statistical validity. This study was conducted in accordance with the Declaration of Helsinki and approved by the Ethics Committee of the Prefectural University of Hiroshima (Approval No. 20HH005). Written informed consent was obtained from all participants.

Test meals

Two test conditions were established: CR and RP. To ensure consistency, commercially available pre-packaged products were used: Koshihikari Komori 150 g (Sato Foods Co., Ltd., Niigata, Japan) for CR and Zen-gayu 200 g (Holica Foods Co., Ltd., Niigata, Japan) for RP. Carbohydrate content was standardized to 40.7 g for both meals. The CR meal consisted of 101 g of CR served with 16 g of savoury sea bream miso (Tai-miso; Mishima Foods Co., Ltd., Hiroshima, Japan), whereas the RP meal comprised 300 g of RP served with 16 g of the same Tai-miso.

Nutritional components were calculated based on the Standard Tables of Food Composition in Japan (Table 1). The preparation methods for the test meals strictly followed the instructions provided on the commercial product packages. The cooked white rice was reheated in a microwave oven at 600W for one minute and 40 seconds before consumption. The RP was consumed without reheating, as no cooking instructions were provided on its packaging. To minimize weighing errors, identical digital kitchen scales (KJ-222; Tanita Corporation, Tokyo, Japan) provided by the researchers were distributed to the participants, who then weighed the portions themselves.

**Table 1:** Nutritional content of test meals.

	Cooked rice (101 g)	Rice porridge (300 g)	Tai-miso (16 g)
Energy (kcal)	140.7	136.5	46
Protein (g)	1.95	2.4	0.8
Fat (g)	0	0	0.4
Carbohydrates (g)	32.9	31.8	9.6
Available carbohydrate (g)	31.5	31.5	9.2
Dietary fiber (g)	1.34	0	0.4
Salt equivalent (g)	0	3	0.5

Study design

A randomized, controlled crossover design was employed. Subjects were assigned to either the CR-first or RP-first sequence using the RAND function in Excel (Microsoft Corp., Redmond, WA, USA). A washout period of at least one day was mandated between the two sessions to eliminate the "second-meal effect" and potential carry-over effects from consecutive testing [6,7].

Measurement of IFG

IFG levels were monitored using the FreeStyle Libre system. A sensor was applied to the back of the non-dominant upper arm. Testing began at least 24 hours after sensor application to allow for stabilization. Participants were prohibited from consuming anything except water for eight hours prior to the test meal consumption and for two hours post-consumption. Furthermore, participants were required to comply with remaining at rest and avoiding exercise for two hours immediately following the test meal ingestion. Subjects consumed the assigned meal within 10 minutes.

To simulate poor bolus formation due to impaired mastication, subjects were strictly instructed to limit chewing to 10 strokes per mouthful. Following ingestion, subjects remained sedentary for 120 minutes, during which only water intake was permitted. IFG was recorded at 0 (baseline), 15, 30, 45, 60, 75, 90, 105, and 120 minutes. Compliance was verified by analyzing the continuous data uploaded from the devices post-study.

Evaluation of subjective fullness

Subjective fullness was assessed using a 100-mm Visual Analogue Scale (VAS) at 0 and 120 minutes post-ingestion.

Statistical analysis

Data were analyzed using RStudio (Posit, Boston, MA, USA). Temporal changes in postprandial blood glucose levels were evaluated using a two-way repeated measures analysis of variance (ANOVA), followed by post hoc pairwise comparisons with estimated marginal means for individual time points. The primary endpoint was the incremental area under the curve (iAUC 0-120 min) for IFG. Secondary endpoints included time-specific IFG levels, iAUC at different intervals (0-30, 0-60, 0-90 min), and VAS scores. A p-value of < 0.05 was considered statistically significant.

## Results

Participant characteristics

A total of 38 participants were initially recruited. Two participants were excluded based on the exclusion criteria, and five were excluded due to non-compliance with the study protocol (e.g., failure to adhere to the 10-minute consumption limit or incomplete glucose monitoring). Consequently, data from 31 participants (mean age: 20.3 ± 1.2 years) were included in the final analysis. The demographic data, including height, weight, and BMI, are summarized in Table 2.

**Table 2:** Demographic characteristics of the study participants (n = 31).

Characteristic	Mean ± SD
Age (years)	21.4 ± 1.0
Height (cm)	159.9 ± 5.9
Weight (kg)	52.6 ± 7.3
Body mass index (kg/m^2^)	20.6 ± 2.7

Postprandial IFG response

**Figure 1:**
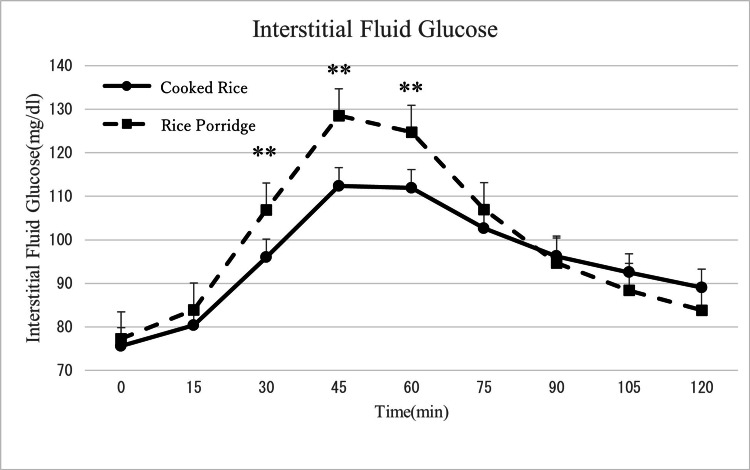
Changes in blood glucose levels following the intake of cooked rice and rice porridge (n = 31). Symbols: ● Cooked rice (101 g rice + 16 g miso), ■ Rice porridge (300 g porridge + 16 g miso). Values are presented as mean ± S.D. Significant differences are indicated by *p < 0.05 and **p < 0.01 using two-way repeated measures analysis of variance (ANOVA).

The mean IFG trajectories for both the CR and RP conditions are shown in Figure 1. Both meals reached peak IFG levels at 45 minutes post-ingestion.

At baseline (0 min), no significant difference in IFG was observed between the CR group (75.6 ± 11.6 mg/dL) and the RP group (77.3 ± 10.8 mg/dL) (p = 0.611), with a mean difference of -1.6 mg/dL (95% CI: -29.1 to 25.8).

However, after reaching the peak, the RP group showed significantly higher IFG values compared with the CR group at 30, 45, and 60 minutes (p = 0.008, p < 0.001, and p < 0.001, respectively). No significant differences were observed at other time points.

Compared with the CR group, the RP group showed significant differences at the 0-60, 0-90, and 0-120 minute intervals (p = 0.0009, p < 0.001, and p = 0.0054).

Incremental area under the curve (iAUC)

**Figure 2:**
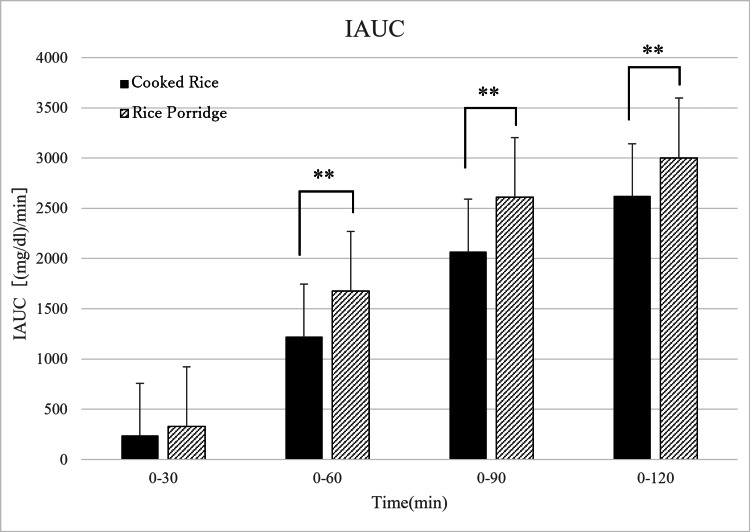
Incremental area under the curve (iAUC) for blood glucose after consumption of cooked rice and rice porridge (n = 31). ■ Cooked rice: 101 g of cooked rice with 16 g of sea bream miso; ▨ Rice porridge: 300 g of rice porridge with 16 g of sea bream miso. Values are presented as mean ± S.D. *p < 0.05, **p < 0.01 vs. cooked rice (or between groups) by two-way repeated measures analysis of variance (ANOVA).

Compared to the CR group, the RP group showed a significantly higher iAUC at all time points (Figure 2). Specifically, significant differences were observed at 0-30 and 0-120 min (p < 0.05), while differences at 0-60 and 0-90 min were highly significant (p < 0.01).

Subjective fullness

Subjective fullness was analyzed for 28 participants (excluding three with missing data). At baseline (0 min), fullness scores were 0 mm for both conditions. At 120 minutes post-ingestion, the mean VAS scores were 3.1 ± 2.3 mm for the RP group and 1.8 ± 3.5 mm for the CR group. While the RP group tended to feel slightly fuller, the difference was not statistically significant (p = 0.1).

Specifically, significant differences were observed at 0-30 and 0-120 min (p < 0.05), while differences at 0-60 and 0-90 min were highly significant (p < 0.01).

Subjective fullness

Subjective fullness was analyzed for 28 participants (excluding three with missing data). At baseline (0 min), fullness scores were 0 mm for both conditions. At 120 minutes post-ingestion, the mean VAS scores were 3.1 ± 2.3 mm for the RP group and 1.8 ± 3.5 mm for the CR group. While the RP group tended to feel slightly fuller, the difference was not statistically significant (p = 0.1).

## Discussion

Effect of food form on postprandial glycemic response

The present study demonstrated that RP induces a significantly higher postprandial IFG response compared to CR, despite identical carbohydrate content. This was evidenced by higher IFG levels at 30, 45, and 60 minutes and a consistently larger iAUC across all time intervals (p < 0.05). The rapid rise in IFG in the RP group can be attributed to several physicochemical factors.

There is a substantial difference in the cooking methods between RP and standard CR; it is well-established that standard cooking protocols for porridge involve significantly higher water-to-rice ratios and extended heating durations. Consequently, the physical structure of RP is structurally weaker or more disrupted compared to that of CR.

Prior literature demonstrates that increased water volume and prolonged heating intervals cause the disruption and collapse of starch granules. Smaller granule sizes lead to enhanced water absorption capacity, higher solubility, greater swelling power, and an increased susceptibility to gelatinization [5,10-12]. Starch gelatinization involves irreversible transitions, including the cleavage of hydrogen bonds within the starch granules and an increase in starch solubility [13]. This advanced state of gelatinization facilitates a higher rate of starch digestion by α-amylase, thereby making postprandial blood glucose levels more prone to rapid elevation [5,13,14].

Therefore, it is highly probable that the sharper postprandial glucose rise observed in the porridge group was driven by the alterations in granule size and the accelerated gelatinization resulting from the greater water content and longer cooking time inherent to standard porridge preparation. Although the precise microstructural mechanics of glucose elevation could not be empirically isolated in the present study, our findings strongly suggest that the postprandial glucose response to Japanese rice varies substantially depending on the specific cooking methodology. Specifically, longer cooking durations for porridge appear to increase its glycemic impact. In Japan, traditional porridge-making methods vary widely, ranging from cooking raw polished rice directly in excess water to simmering previously CR. Future investigations are warranted to clarify the precise extent to which these methodological variations in cooking time and water volume influence subsequent postprandial glycemic profiles.

Subjective fullness and energy density

Interestingly, no significant difference was observed in subjective fullness at 120 minutes post-ingestion (p = 0.1), although the RP group (300 g) had nearly three times the physical volume of the CR group (101 g). This lack of difference may be explained by the lower energy density of porridge (0.5 kcal/g) compared to CR (1.4 kcal/g). According to previous research, reductions in portion size and energy density can have additive effects on satiety [15]. In our study, the increased gastric distension from the larger volume of porridge may have been offset by its faster gastric emptying and lower caloric concentration, resulting in a similar satiety level to CR after two hours.

Clinical implications for nutritional management

RP is often preferred and frequently consumed in clinical settings because it is safe and easy to swallow. Incorporating lipids, proteins, or dietary fiber into meals is well known to delay gastric emptying and suppress postprandial blood glucose elevation [16,17]. Moving forward, we aim to investigate whether co-ingestion with these nutritional components can suppress the postprandial glycemic rise when consuming a full RP. Furthermore, utilizing thickening agents or specific rice varieties with a low GI could serve as a balanced approach to achieve both swallowing safety and metabolic control.

Limitations

Several limitations of this study must be acknowledged. First, the study population primarily consisted of healthy young adults, and the majority were females. While this gender imbalance was a point of concern in previous reviews, we believe the results remain valid due to our randomized crossover design, where each participant served as their own control. This minimizes the impact of inter-individual physiological differences (e.g., hormonal status or muscle mass) on the comparison between CR and RP. Second, mastication was artificially limited to 10 strokes, which may not fully represent the natural eating behavior of all individuals, although it effectively simulated impaired oral processing. Third, IFG was measured using the FreeStyle Libre system, which exhibits a physiological time lag of 5-10 minutes compared to venous blood glucose [18]. Finally, we did not measure insulin, incretins (glucagon-like peptide-1 (GLP-1) and glucose-dependent insulinotropic polypeptide (GIP)), or gastric emptying rates, limiting our ability to confirm the exact hormonal mechanisms.

There are several limitations to this study. Another limitation is that this study was a relatively small-scale, single-center trial, and the participant cohort was predominantly female. To evaluate the impact of sex on the glycemic outcomes, an additional analysis using a linear mixed-effects model was conducted. The model revealed significant main effects for food (p < 0.001) and time (p < 0.001), as well as a significant food × time interaction (p = 0.013). In contrast, no significant main effect of sex (p = 0.0993) or sex-related interactions was observed (sex × food: p = 0.1248; sex × time: p = 0.9768; sex × food × time: p = 0.9777). Post-hoc pairwise comparisons using estimated marginal means demonstrated no significant sex differences at any individual time point during rice consumption (p > 0.05). However, during porridge consumption, a significant sex difference was observed exclusively at 120 minutes, where females exhibited significantly lower glucose levels than males (estimate: -22.47 mg/dL, p = 0.0134).

Although no broad, gender-driven confounding effects were statistically observed except for the specific 120-minute point of porridge, the possibility that the study was underpowered to detect subtle sex differences across all time points cannot be entirely ruled out due to the small scale (27 females and four males). Therefore, caution should be exercised when generalizing these findings. Future multi-center studies with larger and more gender-balanced cohorts are warranted to validate our results.

Second, individual variations in the implementation of the methodology represent a limitation. Because this study was conducted at the participants' homes, where they prepared and consumed the test meals independently, variability may have occurred in the accuracy of meal weight and adherence to the mastication restriction. The distribution of identical kitchen scales minimized errors in meal weight. The lack of objective monitoring for the number of chews leaves the exact level of adherence unclear. Additionally, it remains uncertain to what extent a 10-stroke mastication process in healthy adults replicates the characteristics of the bolus formed in the oral cavity of patients with impaired masticatory and swallowing functions. Therefore, future studies with rigorous ethical considerations are warranted to investigate whether consistent results can be obtained in individuals with reduced masticatory and swallowing functions.

Third, the functional limitations of the measurement apparatus must be acknowledged. Postprandial glycemic responses were monitored using the FreeStyle Libre system, which introduces a physiological lag of 5-10 minutes compared with venous blood glucose measurements [18].

Finally, our ability to elucidate precise hormonal mechanisms was constrained because insulin, incretin hormones (GLP-1 and GIP), and gastric emptying rates were not measured.

## Conclusions

In conclusion, RP induces a more rapid and higher postprandial glycemic response than CR in healthy young adults under restricted mastication. These results emphasize that food form is as critical as carbohydrate quantity in managing postprandial glycemia. Future research should investigate these responses in elderly populations with diagnosed diabetes and explore the effectiveness of various food additives (e.g., lipids or thickeners) in stabilizing glucose levels without compromising swallowing safety.
